# Exploiting Phagocytic Checkpoints in Nanomedicine: Applications in Imaging and Combination Therapies

**DOI:** 10.3389/fchem.2021.642530

**Published:** 2021-03-03

**Authors:** Madeleine R. Landry, Joshua M. Walker, Conroy Sun

**Affiliations:** ^1^ Department of Pharmaceutical Sciences, College of Pharmacy, Oregon State University, Portland, OR, United States; ^2^ Department of Radiation Medicine, School of Medicine, Oregon Health & Science University, Portland, OR, United States; ^3^ Department of Cell, Developmental, and Cancer Biology, School of Medicine, Oregon Health & Science University, Portland, OR, United States

**Keywords:** immunotherapy, nanoparticles, drug delivery, contrast agent, don’t eat me, eat me

## Abstract

Recent interest in cancer immunotherapy has largely been focused on the adaptive immune system, particularly adoptive T-cell therapy and immune checkpoint blockade (ICB). Despite improvements in overall survival and progression-free survival across multiple cancer types, neither cell-based therapies nor ICB results in durable disease control in the majority of patients. A critical component of antitumor immunity is the mononuclear phagocyte system and its role in both innate and adaptive immunity. The phagocytic functions of these cells have been shown to be modulated through multiple pathways, including the CD47-SIRPα axis, which is manipulated by cancer cells for immune evasion. In addition to CD47, tumors express a variety of other “don’t eat me” signals, including beta-2-microglobulin and CD24, and “eat me” signals, including calreticulin and phosphatidylserine. Therapies targeting these signals can lead to increased phagocytosis of cancer cells; however, because “don’t eat me” signals are markers of “self” on normal cells, treatment can result in negative off-target effects, such as anemia and B-cell depletion. Recent preclinical research has demonstrated the potential of nanocarriers to synergize with prophagocytic therapies, address the off-target effects, improve pharmacokinetics, and codeliver chemotherapeutics. The high surface area-to-volume ratio of nanoparticles paired with preferential size for passive targeting allows for greater accumulation of therapeutic cargo. In addition, nanomaterials hold promise as molecular imaging agents for the detection of phagocytic markers. This mini review highlights the unique capabilities of nanotechnology to expand the application and efficacy of immunotherapy through recently discovered phagocytotic checkpoint therapies.

## Introduction

**FIGURE 1:**
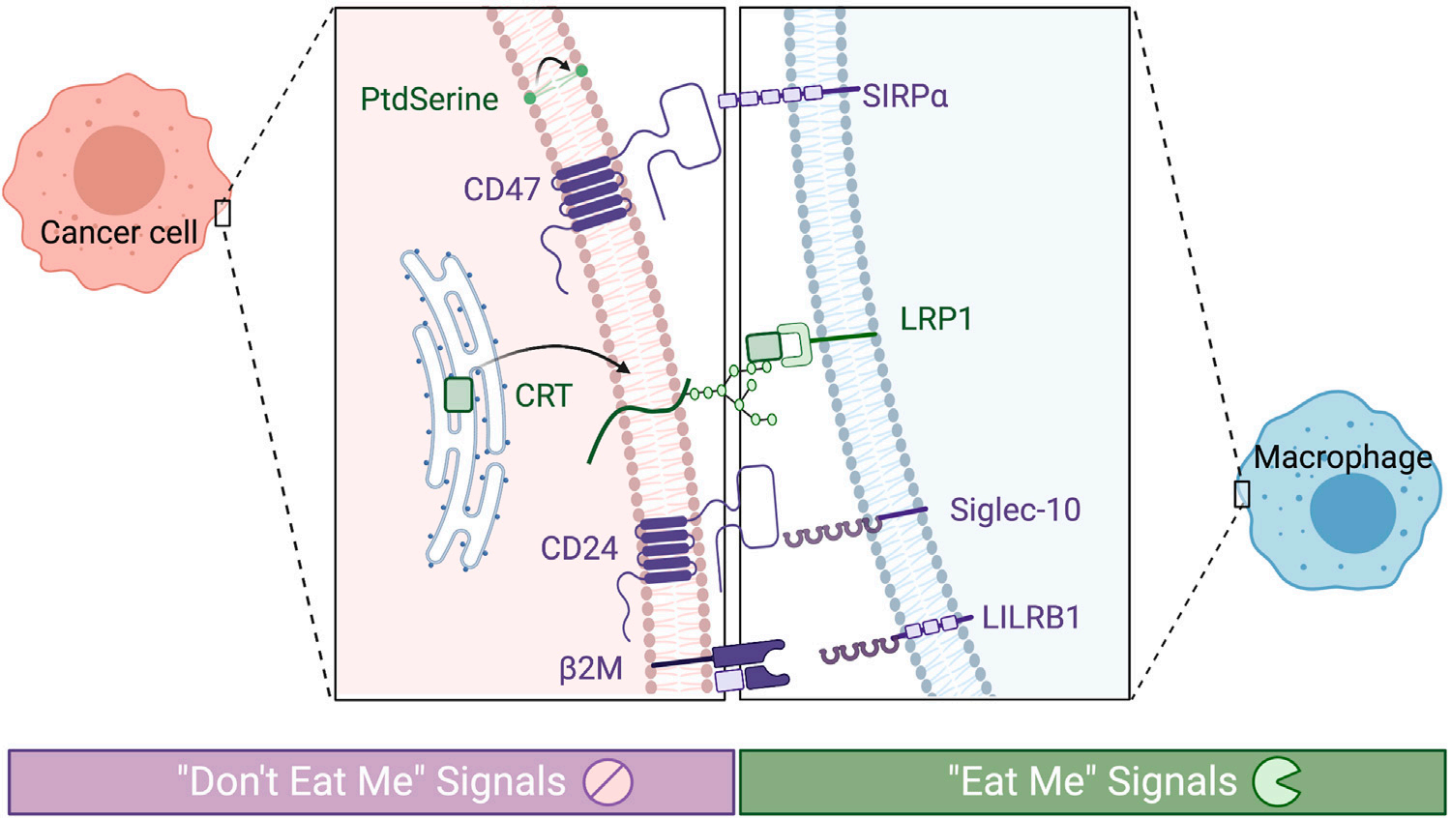
Phagocytic checkpoints can be grouped by prophagocytic signals (“eat me,” in green) or antiphagocytic (“don’t eat me,” in purple). Known receptors expressed on tumor associate macrophages are depicted on the right and their corresponding signals on the cancer cell on the left.

Under normal circumstances, the body relies on a functioning innate immune system to rapidly respond to cues for phagocytosis. This process includes detection of pathogen invasion, clearance of apoptotic and necrotic cellular debris, and the processing and presentation of foreign or tumor antigens. Critical molecular markers of phagocytosis, or “eat me” signals, trigger engulfment by members of the mononuclear phagocyte system (see Figure 1) (Ravichandran, 2011; Li, 2012; Li et al., 2018; Feng et al., 2019). In healthy cells, the expression of these signals increases as they age to recruit phagocytes to clear them, while malignant cells can downregulate the expression of these markers to evade phagocytosis and cell death. Normal healthy cells also rely on antiphagocytic “don’t eat me” markers that identify the cell as “self” and prevent premature phagocytosis. By both suppressing “eat me” and amplifying “don’t eat me” signals, cancer cells have been demonstrated to evade destruction by phagocytic cells and the resulting adaptive immune response which they stimulate. The potential benefit of controlling phagocytic checkpoints for lasting treatment response has been an area of significant research recently. Targeting immune-modulating pathways present on antigen-specific T-cells has been successful and resulted in substantial clinical advances (CTLA-4, PD-1); however, there is growing evidence that upregulation of antigen presentation and the direct antitumor effects of macrophage and dendritic cells through prophagocytic therapy will further improve outcomes. As a result, prophagocytic therapies have been proposed as an adjuvant treatment, alongside chemotherapy, radiation, and/or immunotherapy (Feng et al., 2019). Administration of multiple treatment modalities complicates the dosing schedule and compromises patient adherence. Here, next-generation drug carriers that allow for delivery of multiple therapies and controlled spatiotemporal release may simplify dosing to enable these advanced treatment regimens. In particular, nanotechnology-based strategies for the delivery of phagocytic regulating therapy may address these challenges, along with other shortcomings of checkpoint blockade.

Nanotechnology offers solutions to fundamental issues with systemic delivery of immunotherapies (Irvine and Dane, 2020). Perhaps most importantly, nanocarriers can increase drug localization—by both passive and active targeting—that increases safety by preventing off-target effects (antigen sink), allowing for lower, more effective doses. One of the initial rationales for the use of nanomedicine in cancer therapy was a favorable size that allowed for higher accumulation within tumor vasculature because of the enhanced permeability and retention (EPR) effect. However, this phenomenon and the enhancement of drug accumulation in tumors are highly debatable (Wilhelm et al., 2016; Dai et al., 2018; Ouyang et al., 2020). Despite this controversy, nanotechnologies have much to offer as drug carriers and therapeutic biomaterials. First, nanoparticles (NPs) have a high surface area-to-volume ratio, making them ideal for coating with high-affinity ligands, i.e., targeting agents. Second, they can serve as a depot for the payload they are delivering and allow for controlled release that is pH-specific or triggered by external stimuli (Irvine et al., 2015). Third, NPs are well suited to act as theranostic agents, with sensing capabilities to exploit the recent discovery of “eat me” and “don’t eat me” signals differentially expressed on cancer tissue compared to normal tissues (Li et al., 2020; Garg et al., 2016). Nanomaterials are also capable of influencing immune response based on size, charge, shape, and hydrophobicity, in addition to carrying immunomodulating cargo or enhancing immunomodulation upon external stimuli.

Nanomaterials may also serve as molecular imaging probes to monitor immune-related markers by conventional medical imaging modalities, such as magnetic resonance imaging (MRI) and positron emission tomography (PET) imaging (Choi et al., 2020). Combined imaging and delivery platforms have been developed with multifunctional nanomaterials for immune-related applications. For instance, Xu et al. synthesized mesoporous silica NPs for theranostic PET-guided photodynamic therapy and neoantigen-based cancer vaccination (Xu et al., 2019). Iron oxide NPs have also been found to be taken up by tumor-associated macrophages, which can then provide MRI imaging of phagocytic activity (Mohanty et al., 2019a). Furthermore, nanomaterials can capitalize on the inherent ability of these imaging modalities and treatment modalities, such as radiation or photodynamic/thermal therapy (PDT and PTT), to induce an immune response (Zhang et al., 2020a). NPs formulated from high-Z elements and reactive oxygen species (ROS) generating metals can enhance the ROS generation when stimulated by radiation (Ni et al., 2018; Neufeld et al., 2019; Choi et al., 2020; Ni et al., 2020). This ROS production results in endoplasmic reticulum (ER) stress and both calreticulin (CRT) and phosphatidylserine (PS) exposure, which are potent “eat me” signals and danger-associated molecular patterns (DAMPs). Nanoparticle-enhanced PTT and PDT can likewise produce increased ROS with potential immunological response (Tada and Baptista, 2015; Gao et al., 2020).

## “Don’t Eat Me” Signals: CD47, Beta-2-Microglobulin, and CD24

### CD47

CD47 was the first phagocytic checkpoint to be identified and the first with therapies in phase 2 clinical trials (Majeti et al., 2009; Advani et al., 2018b). The expression of CD47, which is a transmembrane protein, decreases in red blood cells as they age; this “self” marker has also been found to be upregulated in the majority of cancers. CD47 interacts with inhibitory receptor signal regulatory protein alpha (SIRPα) on myeloid cells, preventing phagocytosis. Disruption of the axis stimulates antigen-presenting cells (APC)—specifically CD8+/CD103 + dendritic cells—and cross-presentation of tumor antigen, resulting in priming or reactivation of tumor-specific T-cell immunity. CD47 therapies range from blocking antibodies, such as Hu-5F9 (an anti-CD47 antibody), to blocking peptides and high-affinity monomers for SIRPα (Liu et al., 2004; Weiskopf et al., 2013; Sikic et al., 2019). These therapies largely rely on CD47 blockade paired with a second therapy that stimulates adaptive immunity, such as traditional chemotherapy and/or immunogenic cell death (ICD)- inducing chemotherapies (Advani et al., 2018a; Fisher et al., 2020). In patient-derived xenograft models, CD47 blockade as monotherapy has proven unsuccessful in regard to tumor volume control and durability (Chao et al., 2010; Cioffi et al., 2015). However, combined with other therapies, CD47 blockade is able to enhance tumor volume control and extend response to treatment. Both in research and clinically, CD47 therapies have been limited by hematologic toxicity (anemia and hemagglutination) (Sikic et al., 2019). The need for a multimodal approach and reduction of off-target side-effects makes NPs a promising solution as carriers for both CD47 blockade and chemotherapy.

In preclinical models, CD47 nanomedicines have exploited CD47 upregulation both for cell targeting to increase drug delivery and as an innate immune checkpoint. Multiple pro- and antiphagocytic signals play a role in the endocytosis of tumor cells and NPs can be beneficial as codelivery agents of two signal modulators, such as to suppress CD47–SIRPα interactions and enhance CRT presence at the tumor (Zhang et al., 2020b). Zhang et al. used copper-free click-chemistry to conjugate CD47 antibodies and CRT onto the surface of azide-modified silica NPs. Similarly, Ramesh et al. co-loaded inhibitors of colony-stimulating factor 1 receptor inhibitor (to prevent macrophage polarization to the protumorigenic phenotype) and Src homology region 2 domain phosphatase (activated downstream of CD47–SIRPα) into a lipid NP (Ramesh et al., 2019). They found that the co-loaded NP gave superior phagocytic capabilities compared to individual drug treatments and minimal effects from the free drug at later timepoints. Additionally, CD47-coated NPs can act as stealth coatings, taking advantage of CD47 as a marker of self (Qie et al., 2016; Song et al., 2019). Qie et al. modified the surface of polystyrene beads with polyethylene glycol or CD47 and found CD47 is able to lower the phagocytic activity of classically activated macrophages (Qie et al., 2016). Song et al. similarly utilized the stealth coating by synthesizing nanoscale artificial antigen-presenting cells (aAPC) to expand antigen-specific T-cell populations (Song et al., 2019). Nanoscale aAPC offer favorable biodistribution and reduced embolism compared to conventional aAPC.

Recently, CD47 nanobodies have been effective at addressing the toxicity limitation of anti-CD47 treatment (Ma et al., 2020). Nanobodies, or single domain antibody fragmentations, have been shown to reduce agglutination of RBC’s (Ma et al., 2020) and synergize with PD-L1 therapy (Sockolosky et al., 2016; Ingram et al., 2017).

### Beta-2-Microglobulin

β2-Microglobulin (β2M) is a glycoprotein that functions as the light-chain component of MHC-I and plays a critical role in the thymic selection and host–pathogen interaction (Bevan, 2010). β2M has been considered a therapeutic target in cancer due to the activation of NK cells in β2M-deficient models. Treatment with anti-β2M antibodies can result in the release of proinflammatory cytokines. Recently, β2M has been demonstrated to interact with inhibitory receptor leukocyte immunoglobulin-like receptor subfamily B member 1 (LILRB1) to prevent phagocytosis. Barkal et al. found that cancer cells with higher levels of MHC class I proteins or upregulated LILRB1 on tumor-associated macrophages (TAMs) were not responsive to anti-CD47 therapy and had lower levels of phagocytosis (Barkal et al., 2018). Like CD47, MHC-I is expressed ubiquitously, resulting in a need for tumor targeting to prevent unwanted adverse effects. However, β2M has been found to be elevated in multiple myeloma, lymphoma, and prostate cancer patients and is a prognostic marker (Mink et al., 2008; Koelzer et al., 2012; Miyashita et al., 2015).

Currently, there are limited nano-based applications for β2M. However, since β2M has been identified as a prognostic marker for certain cancers, nanosensors have been proposed as a noninvasive method of detection. Rizwan et al. reported the development of a highly sensitive (fg ml^−1^) label-free electrochemiluminescence immunosensor for detecting β2M in serum and urine (Rizwan et al., 2017). This sensor was composed of a CdSe quantum dot screen-printed electrode modified with gold NPs doped with a carbon nano-onion chitosan nanocomposite. This nanoplatform was developed for renal disfunction prior to the discovery of β2M’s role as a “don’t eat me” signal, and it was not originally intended to be used in cancer therapy. However, this application may be well suited for the detection of β2M in the serum of cancer patients with elevated β2M serum levels, which have been reported in prostate cancer, Hodgkin lymphoma, and diffuse large B-cell lymphoma patients. Others have used gold NPs as sensors for β2M as well, coating the gold NPs with anti-β2M antibodies and forming a stable water suspension (Maity et al., 2020). When we consider the high sensitivity of the sensors and biocompatibility, their application could potentially serve as a noninvasive method compared to biopsies. Gold NPs are ideal as sensors for β2M and other phagocytic checkpoints because they can be easily conjugated with antibodies, nucleic acids, and other targeting agents.

### CD24

CD24 (heat-stable antigen) is a cell surface glycosylphosphatidylinositol-anchored protein expressed widely on various cell types, including hematopoietic T- and B-cells and APCs, nonhematopoietic cells, and cancer cells (Fang et al., 2010). CD24 is involved in inflammation with roles as a costimulatory for T-cell activation in lymphoid organs and in mediating apoptosis signaling (Suzuki et al., 2001; Fang et al., 2010). CD24 is also responsible for distinguishing between DAMPs and pathogen-associated molecular patterns (PAMPs) via the interactions with sialic acid-binding Ig-like lectin 10 (Siglec-10) (Liu et al., 2009). Recently, CD24 was discovered as a “don’t eat me” signal that interacts with Siglec-10 on TAMs to circumvent phagocytosis. This mechanism was elucidated by Weissman and colleagues (Barkal et al., 2019). CD24 (or heat-stable antigen) is a heavily glycosylated GPI-anchored surface protein that is well-known for other modes of action to dampen immune response (Barkal et al., 2019). Barkal et al. found that tumors refractory to CD47 treatment are often responsive to CD24 blockade. Some tumors respond to neither, indicating that there are likely other phagocytic checkpoints regulating macrophage response. In addition, others have found that upregulation of CD24 is a poor prognostic factor for many cancer types (Kristiansen et al., 2004; Yang et al., 2009; Kwon et al., 2015; Wang et al., 2018). This is an area of opportunity in which nanomaterials would be well suited as an agent to both target and monitor CD24 expression, combined with blockade. Prior to the discovery of CD24 as a “don’t eat me” signal, docetaxel-loaded PLGA-PEG NPs conjugated with anti-CD24 were found to have a 10-fold higher prostate tumor accumulation in mice and showed potential for CD24-tagged NPs as an imaging agent (Bharali et al., 2017). Barkal et al. also found that CD24 does not undergo an FcR-dependent route of phagocytosis, indicating that nanobodies would be well suited for CD24 therapies.

## “Eat Me” Signals: Calreticulin and Phosphatidylserine

### Calreticulin

Calreticulin is a membrane-anchored “eat me” signal that is usually conserved in the ER. Under conditions of cell ER stress, CRT is translocated to the exterior of the cell membrane. CRT exposure triggers dendritic cell uptake and has recently been used as a marker for ICD. CRT release acts as a DAMP that can trigger cytokine release.

NP-based therapies can induce CRT exposure by the material themselves or by serving as carriers of ICD-inducing drugs (Landry et al., 2020). Ni et al. utilized hafnium metal-organic frameworks (MOFs) in CT26 cells to stimulate CRT exposure and induce an immune response (Ni et al., 2018). The MOFs themselves led to the expression of CRT on the cell surface, which was further enhanced by radiation. Nanomaterials may offer the advantage of synergizing with other treatment modalities, such as radiation and ultrasound, in a localized manner. These modalities alone are able to increase CRT exposure as well, further contributing to phagocytosis. Sethuraman et al. found that encapsulating a CRT plasmid in a liposome and delivering with focused ultrasound (FUS) was sufficient to modulate the CRT-CD47-PD-L1 axis (Sethuraman et al., 2020). Specifically, they found that delivering the CRT nanoparticle alone increased the surface exposure of CRT twofold but also increased “don’t eat me” signal CD47 in B16F10 melanoma cells. However, when they delivered the CRT nanoparticle with FUS heating, they found a 3-fold increase in CRT and no significant change in CD47. Furthermore, *in vivo*, they found melanoma-specific immunity and an increased PD-1/PD-L1 expression in T-cells with the combination. Others have similarly applied different forms of targeted thermal therapy. Li et al. targeted PDT/PTT to the ER to directly induce CRT exposure and inflict ICD (Li et al., 2019). They localized the therapy to the ER by ER-specific pardaxin peptide modified indocyanine green conjugated-hollow gold nanospheres with an oxygen-delivering hemoglobin liposome. Using the NP and external near IR light, they could effectively control and monitor treatment.

### Phosphatidylserine

Phosphatidylserine (PS) is a negatively charged lipid usually constrained to the inner leaflet of the cell membrane. During apoptosis, PS is translocated to the outer leaflet, marking cells for uptake by macrophages and other APCs. In cancer, the membrane integrity is likewise disrupted, and phosphatidylserine is flipped to the outer leaflet. While PS acts as an “eat me” signal by sending out abnormal signals that macrophages can detect, it is also immunosuppressive by inducing macrophage polarization from the proinflammatory M1 phenotype to the protumor M2 phenotype, resulting in secretion of anti-inflammatory cytokines IL-10 and TGF-β (Birge et al., 2016). PS has long been recognized as a promising imaging target (Bagalkot et al., 2016; Chang et al., 2020). Annexin V is commonly used to stain phosphatidylserine as an apoptosis marker. Prior to discovery as an “eat me” signal, Thorpe and colleagues reported on PS as a marker of tumor vasculature with expression ranging from 4 to 40% of cancer vessels in six different tumor types. Additionally, they found hypoxia/reoxygenation, acidity, inflammatory cytokines, thrombin, or hydrogen peroxide-induced PS exposure on cultured endothelial cells (Ran and Thorpe, 2002). Advanced cancer states promote this environment, while many treatment modalities also further induce these conditions, indicating PS as an ideal target.

Recent interest in targeting PS with nanoparticles has largely involved saposin C (SapC), a lysosomal protein with an affinity for PS. Chu et al. formulated stable nanovesicles composed of SapC and dioleoylphosphatidylserine (DOPS), which were also labeled with CellVue Maroon dye for fluorescent microscopy and fluorescence imaging (Qi et al., 2009). SapC showed high-coupling efficiency with DOPS and was effectively incorporated into the lipid bilayer with a facile sonication-based synthesis. Saposin is ideal for PS targeting in a tumor as it preferentially interacts with unsaturated, negatively charged lipids in an acidic environment. When applied in an orthotopic pancreatic cancer mouse model, extended survival and enhanced nanoparticle accumulation were observed in the tumor for four days (vs. clearance from the liver within 24 h) (Chu et al., 2013). More recently, Davis et al. applied the same nanovesicle system to radiation-treated tissue (Davis et al., 2019). As mentioned previously, radiation enhances the surface exposure of certain “eat me” signals, including PS and CRT. This phenomenon can be capitalized on by locally delivering radiation prior to PS-targeting NPs to further selectively target tumors. Davis et al. found that cancer cells with low expression of PS had a more pronounced increase in PS exposure after irradiation than cells with low PS exposure. Additionally, they found that radiation-induced increase in surface PS is both dose- and time-dependent. Zhang et al. likewise used radiation to prime the cells with PS. They designed a SapC-containing liposomal nanoprobe composed of PEG-coated nanoparticles conjugated with a human mAb for PS. The NP was designed for delivery after irradiation of a breast cancer tumor (Zhang et al., 2014). The core also contained an MR contrast agent, superparamagnetic iron oxide NPs, and the bilayers of the liposomes were loaded with near-infrared dye. With these nanoparticles, the investigators were able to longitudinally monitor changes in tumor contrast via dual MRI/optical imaging, revealing enhanced tumor contrast from the anti-PS-tagged NPs.

## Discussion

The role of phagocytic checkpoints has been recognized as an essential component of the cross-talk between malignant cells and the innate immune system. In cancer, tumor cells balance both the suppression of antiphagocytic signals and the enhancement of prophagocytic signals to achieve immune escape. To date, monotherapies targeting these checkpoints have seen minimal success, suggesting these drugs are better suited as combination therapies. Recent reports have indicated that the durable success of ICB is dependent on the ability of the monotherapy or combination therapy to stimulate adaptive immunity (Sockolosky et al., 2016; Feng et al., 2019). More importantly, not all cancers uniformly overexpress or suppress all of these signals and significant variability may exist between malignancies and even individual patients. There are still many unknowns regarding the pathways of phagocytic signals. Interestingly, the expression of certain phagocytic checkpoints has recently been identified as a prognostic factor: CD47 (Majeti et al., 2009), CD24 (Kwon et al., 2015), and β2M (Mink et al., 2008). Though not currently utilized, expression levels of phagocytic checkpoints may potentially provide the same prognostic and predictive value as PD-L1 tumor proportion scores. Furthermore, highly sensitive NP-based contrast agents are ideal for detecting the expression of “don’t eat me” signals on cancer cells despite the ubiquitous expression of “self” markers.

Nanotechnology may be particularly beneficial to phagocytic checkpoint therapy for both 1) altering and 2) imaging the checkpoint (see Table 1). Functional nanomaterials offer a variety of novel applications to alter the checkpoint, such as targeted delivery, immunomodulatory capabilities, co-delivery of therapeutic agents, and synergy with other treatment modalities. Some novel nanoparticles are inherently immune-stimulating based on size, shape, charge, and material. These materials enable combination with other prophagocytic modalities, such as radiation, PDT/PTT, and ultrasound. Furthermore, nanomaterials and nanoscale carriers can act as radiosensitizers used to enhance a myriad of immune-stimulating events upon irradiation, including ROS generation, DNA double- and single-strand breaks, DAMP release/ICD, and CRT/PS exposure on the cell surface. NPs can serve the dual purpose of acting as an imaging probe and enabling tracking of immune cells and checkpoints by ultrasound, PET, and MRI (Kirschbaum et al., 2016; Ou et al., 2020). For instance, ultrasound can be used to both express “eat me” signals that can be targeted by NPs and temporarily disrupt cell-cell junctions, allowing for NP entry. Iron oxide nanoparticles are commonly used for MRI and can be encapsulated in lipid nanoparticles containing “eat me” targeting moieties (Bagalkot et al., 2016). As imaging agents for phagocytic checkpoints, NPs are advantageous due to the selection of nanoscale materials (metal, metal-organic, nanobubbles, and tagged polymers) that are preferentially endocytosed. Additionally, they allow for noninvasive tracking and diagnosis, as compared to surgical resection and histological staining. Altogether, nanomaterials hold potential as theranostic agents for phagocytic checkpoints and have only just begun to be employed for this application. Given the ongoing debate surrounding the efficiency of NP-mediated drug delivery, the applications mentioned herein exploiting phagocytic checkpoint therapy present an alternative path toward realizing the grand potential of cancer nanomedicine. Further development of advanced combination therapies incorporating nanotechnology that target and modulate phagocytic checkpoints is warranted and has the potential to improve tumor immunogenicity and subsequent response to therapy.

**TABLE 1:** Various “eat me” and “don’t eat me” signals, their corresponding receptors, and some of the most recent nanomedicine applications.

Tumor expressed signal	Immune cell receptor	Nanotherapies
“Don’t eat me”		
Cd47	SIRPα	CD47-coated stealth NPs Rodriguez et al., (2013); Qie et al., (2016); Song et al., (2019); Rao et al., (2020)
		Anti-CD47 NPs for blockade Wang and Steinmetz (2019); Zhang et al., (2020a); Zhang et al., (2020b)
		Lipid NPs for CD47 blockade Ramesh et al., (2019)
		Nanobodies Sockolosky et al., (2016); Ingram et al., (2017); Ma et al., (2020)
		NPs for imaging of anti-CD47 Mohanty et al., (2019b)
Β2M	LILRB1	NP immunosensor Rizwan et al., (2017)
CD24	Siglec-10	Anti-CD24-tagged NPs for targeting Bharali et al., (2017)
“Eat me”		
Calreticulin	LRP1	NPs inducing CRT exposure Ni et al., (2018); Li et al., (2019); Landry et al., (2020)
		Calreticulin-containing NPs Sethuraman et al., (2020); Zhang et al., 2020b
Phosphatidylserine	TIM/TAM family, bridging molecules, RAGE, Mertk	Nanovesicles targeting PS Qi et al., (2009); Chu et al., (2013); Davis et al., (2019)
		Nanobubbles for ultrasound Chandan and Banerjee (2018)
